# Tracheotomy does not affect reducing sedation requirements of patients in intensive care – a retrospective study

**DOI:** 10.1186/cc4961

**Published:** 2006-07-10

**Authors:** Denise P Veelo, Dave A Dongelmans, Jan M Binnekade, Johanna C Korevaar, Margreeth B Vroom, Marcus J Schultz

**Affiliations:** 1 Department of Intensive Care Medicine, Academic Medical Center, University of Amsterdam, Meibergdreef 9, 1105 AZ Amsterdam, The Netherlands; 2 Department of Anesthesiology, Academic Medical Center, University of Amsterdam, Meibergdreef 9, 1105 AZ Amsterdam, The Netherlands; 3 Department of Clinical Epidemiology and Biostatistics, Academic Medical Center, University of Amsterdam, Meibergdreef 9, 1105 AZ Amsterdam, The Netherlands; 4 Laboratory of Experimental Intensive Care and Anesthesiology, Academic Medical Center, University of Amsterdam, Meibergdreef 9, 1105 AZ Amsterdam, The Netherlands

## Abstract

**Introduction:**

Translaryngeal intubated and ventilated patients often need sedation to treat anxiety, agitation and/or pain. Current opinion is that tracheotomy reduces sedation requirements. We determined sedation needs before and after tracheotomy of intubated and mechanically ventilated patients.

**Methods:**

We performed a retrospective analysis of the use of morphine, midazolam and propofol in patients before and after tracheotomy.

**Results:**

Of 1,788 patients admitted to our intensive care unit during the study period, 129 (7%) were tracheotomized. After the exclusion of patients who received a tracheotomy before or at the day of admittance, 117 patients were left for analysis. The daily dose (DD; the amount of sedatives for each day) divided by the mean daily dose (MDD; the mean amount of sedatives per day for the study period) in the week before and the week after tracheotomy was 1.07 ± 0.93 DD/MDD versus 0.30 ± 0.65 for morphine, 0.84 ± 1.03 versus 0.11 ± 0.46 for midazolam, and 0.62 ± 1.05 versus 0.15 ± 0.45 for propofol (*p *< 0.01). However, when we focused on a shorter time interval (two days before and after tracheotomy), there were no differences in prescribed doses of morphine and midazolam. Studying the course in DD/MDD from seven days before the placement of tracheotomy, we found a significant decline in dosage. From day -7 to day -1, morphine dosage (DD/MDD) declined by 3.34 (95% confidence interval -1.61 to -6.24), midazolam dosage by 2.95 (-1.49 to -5.29) and propofol dosage by 1.05 (-0.41 to -2.01). After tracheotomy, no further decrease in DD/MDD was observed and the dosage remained stable for all sedatives. Patients in the non-surgical and acute surgical groups received higher dosages of midazolam than patients in the elective surgical group. Time until tracheotomy did not influence sedation requirements. In addition, there was no significant difference in sedation between different patient groups.

**Conclusion:**

In our intensive care unit, sedation requirements were not further reduced after tracheotomy. Sedation requirements were already sharply declining before tracheotomy was performed.

## Introduction

Intubated and mechanically ventilated patients in the intensive care unit (ICU) often need sedation to treat anxiety, agitation and/or pain. Indeed, prolonged translaryngeal intubation is a major source of physical discomfort, for which sedation often is necessary [1-5]. In addition, sedation may be required to facilitate mechanical ventilation (MV). However, sedation leads to a depression of cardiovascular, respiratory and immunological systems, and prolongs the time spent on the ventilator; it can therefore influence outcome in critically ill patients [6-11].

Tracheotomy is a common procedure in mechanically ventilated patients in the ICU, especially in those who have an (expected) prolonged duration of MV [12-15]. Tracheotomy is frequently performed 7 to 21 days after the start of MV [14]. A recent meta-analysis suggests that early tracheotomy shortens the duration of artificial ventilation and length of stay in the ICU [16]. Current opinion is that tracheotomy reduces sedation requirements by facilitating oral and bronchopulmonary toilet and increasing patient comfort [1,17,18]. One recent study described the influence of tracheotomy on the amount of sedation needed in critically ill patients [19]. However, the influence of timing of tracheotomy and differences between subgroups have never been examined.

The objective of this single-center observational study was to determine sedation requirements (amount of sedation used and number of mechanically ventilated patients requiring sedation) in our mixed medical–surgical ICU before and after tracheotomy. In addition, we investigated whether this was influenced by the timing of tracheotomy and patient category, namely acute surgical, elective surgical and non-surgical patients.

## Materials and methods

### Ethical approval, and inclusion and exclusion criteria

The study protocol was approved by the local ethics committee; informed consent was not deemed necessary because of the retrospective observational nature of this study and because the study did not modify diagnostic or therapeutic strategies. In our institution, all patients who receive a tracheotomy are recorded in a tracheotomy database. We used this database to identify patients who required tracheotomy from November 2003 until January 2005 and retrospectively analyzed data for all consecutive cases. Patients who had received a tracheotomy before the present admittance to the ICU were excluded from the study, leaving only patients who received a tracheotomy during their current ICU stay for analysis. All data (see below) were retrieved from the patient data management system (PDMS; Metavision, *i*MD*soft*, Sassenheim, The Netherlands).

### Study location

The data were collected in an ICU at the Academic Medical Center, Amsterdam, The Netherlands. In the Academic Medical Center, which is a large teaching hospital, the ICU is a 28 bed 'closed format' department, in which medical/surgical patients (including cardiothoracic surgery and neurosurgical/neurology patients) are under the direct care of the ICU team. The ICU team comprises eight full-time ICU physicians, eight subspecialty fellows, 12 residents and occasionally one intern. Sedation of patients in our ICU is guided by a standardized protocol. Similarly, MV and weaning follow a protocol. Both protocols are available for all ICU members in paper form and on the local intranet.

### Sedation protocol

**Figure 1:**
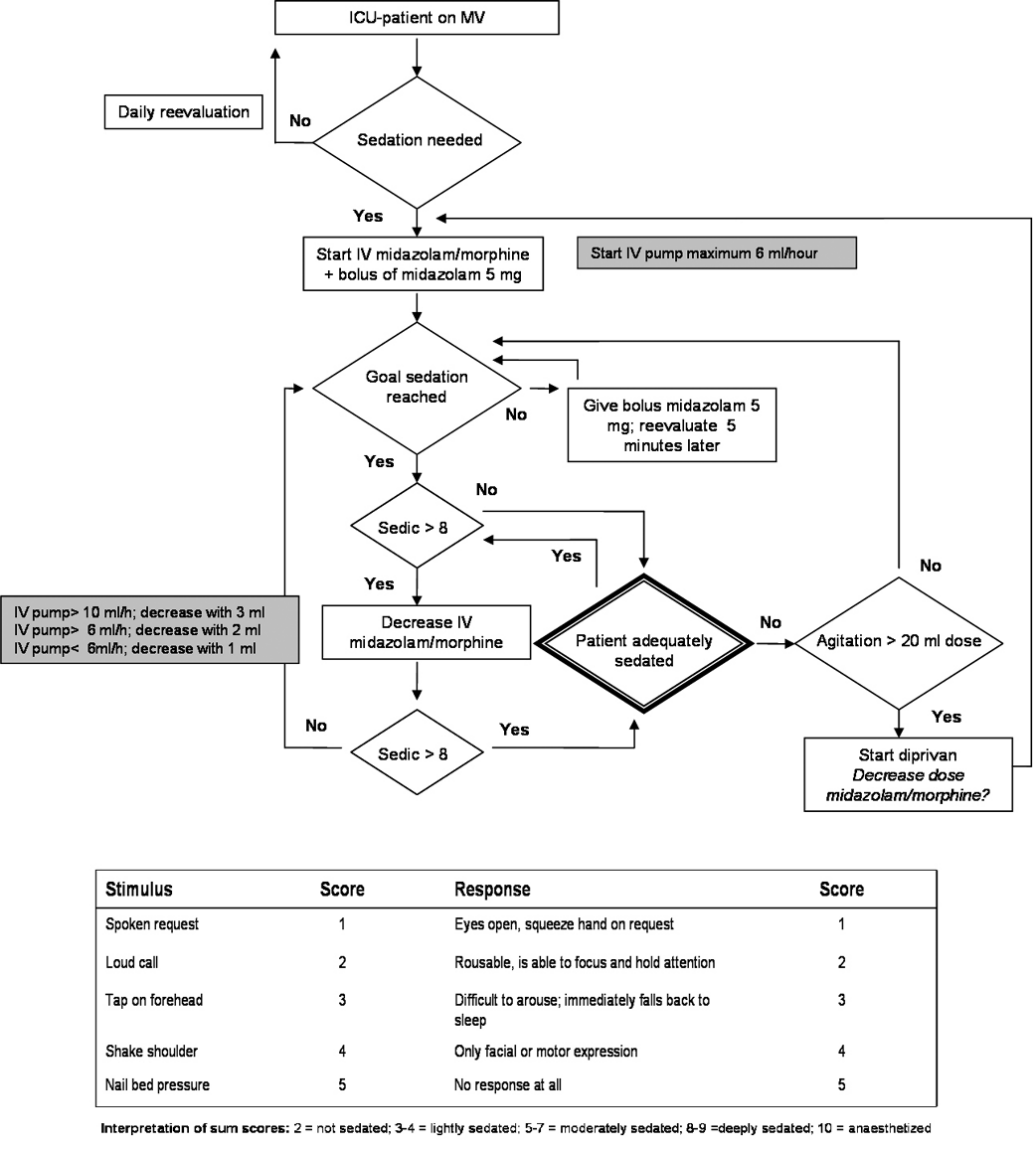
Diagram of the sedation protocol and Sedation Intensive Care (SEDIC) score. IV, intravenous; MV, mechanical ventilation.

In our institution, intravenous sedation usually consists of the combined infusion of morphine and midazolam with 50 ml syringes prefilled with 50 mg of midazolam plus 50 mg of morphine in sterile saline or glucose. Propofol can be used in addition, when high dosages of morphine and midazolam are needed, or solely, when frequent neurological evaluation is warranted (most often only in patients with brain injury). Morphine can also be used separately to control pain when there is no further need for sedation. The goals of sedation are reducing agitation, stress and fear, reducing O_2 _consumption (heart rate, blood pressure and minute volume are measured continuously) and reducing physical resistance and fear for medical examination and daily care. According to the protocol (Figure 1), nurses and physicians determine the level of sedation required each day. Every two hours, the adequacy of sedation for each patient is carefully evaluated by means of a Sedation Intensive Care (SEDIC) score, and the infusion of sedatives is adjusted accordingly [20]. The SEDIC scale consists of five levels of stimuli (from normal speech to nailbed pressure) and five levels of responsiveness (from normal contact to no contact). Sedation levels are defined by the sum of stimulus and response. When a SEDIC score of more than eight is reached, infusion of sedation is reduced. In addition, patients weaned from midazolam receive low-dose oral benzodiazepines (lorazepam and temazepam). Haloperidol is given only to agitated and/or delirious patients.

### Mechanical ventilation protocol

The MV protocol advises pressure-controlled MV or pressure-support MV in all patients; pressure-support MV is started as early as possible. Levels of positive end-expiratory pressure (PEEP) and fraction of inspired oxygen (FiO_2_) are to be adjusted to the arterial partial pressure of oxygen (PaO_2_). In the case of pressure-support MV the level of support is set to reach a respiratory rate at which the patient is breathing comfortably. After tracheotomy, patients start with spontaneous breathing trials when the pressure-support level is less than 15 cmH_2_O and the PEEP level is less than 5 to 7 cmH_2_O. During spontaneous breathing trials, tracheotomy patients are connected to a T piece without a positive pressure valve, through which humidified air with an FiO_2 _of 50% is applied. It is the policy to start with three spontaneous breathing sessions per day. The duration of each session is chosen depending on each patient's condition and indication for tracheotomy: patients who are expected to have normal muscle strength start with more hours per day and, if possible, are kept off the ventilator directly. The number of hours per day is to be increased steadily (by doubling the number of hours over all sessions each day) until complete weaning is reached. Patients are not allowed to breathe spontaneously when haemodynamically unstable, and if patients show desaturation, rapid shallow breathing or signs of fatigue during spontaneous breathing sessions they progress through the protocol more slowly.

### Indications for tracheotomy and procedure

If it is expected that an artificial airway will be needed for less than ten days, translaryngeal endotracheal intubation is preferred. Other than a suspected need for ventilation for more than ten days (protection of the airways), indications for tracheotomy in our unit are repeated failure of weaning from MV, airway obstruction of the upper airways and the need for frequent airway suctioning, for example in patients with critical illness polyneuropathy or low levels of consciousness. Our main contraindications for percutaneous tracheotomy are the following: haemodynamic or pulmonary instability; impalpable trachea (for example due to adiposity); palpable vascular structures or overlying thyroid gland; abnormal anatomy of the neck (for example after surgery, or after radiation); infection of the skin over the trachea. The patients are reassessed on a daily basis to determine whether tracheotomy is required. The decision to proceed with a tracheotomy is made as early as possible; once the decision has been made, it is performed expeditiously unless there is haemodynamic instability, severe coagulopathy, and the need to use high PEEP levels.

In our ICU, as in many institutions in The Netherlands [21], percutaneous tracheotomy is the method of choice; surgical tracheotomy is performed only in those patients in whom percutaneous tracheostomy is contraindicated or fails. In all tracheotomy procedures a Seldinger plus single-step conic dilation technique is used (Ciaglia Blue Rhino; Cook Nederland, Son, The Netherlands).

### Study endpoints

To examine the difference in sedation before and after tracheotomy, we first determined the summed amount of sedatives used during the seven days before and after tracheotomy; in addition, because it better represents the state of the patients around tracheotomy and more accurately describes the need for sedation around that time, we determined the summed amount of sedatives during the two days before and after tracheotomy for each patient. The day of tracheotomy was not included in the analysis because of the expected effect of the tracheotomy procedure on sedation requirements. Because sedation requirements may vary substantially between patients we adjusted for inter-individual variability by dividing the daily dose (DD; the amount of sedatives for each day) by the summed mean daily dose (MDD; the mean amount of sedatives per day for the study period) of sedatives for each patient. For calculation of the MDD the day of tracheotomy was excluded, and because the number of days admitted before and after tracheotomy differed between patients, we corrected for this in the analysis by dividing the summed factor by the number of days. Second, we examined sedation requirements over the days before and after the tracheotomy.

### Influence of timing of tracheotomy and patient category

Because the time between admittance to ICU and the day of tracheotomy differs between patients and may influence sedation needs, we calculated the median time from the start of MV until tracheotomy, and determined the sedation requirements for both 'early' (before median time) and 'late' (after median time) tracheotomy subgroups. In addition, we divided the patients into three groups: non-surgical, acute surgical and elective surgical.

### Statistical analysis

Data were not normally distributed. However, we chose to present the data as means ± standard deviation, because the median was zero and was consequently not informative. Comparisons are made with Wilcoxon signed-rank test for continuous data and the McNemar test for categorical data. Repeated-measurements analysis of variance was used to study changes over time in subjects, taking into account the association between values for individual patients measured at each time point. This implies a maximum of 14 time points per patient. Because the data were not normally distributed we used the logarithm of the DD/MDD for this analysis. However, many measurements of DD/MDD were zero. To solve this problem we added 0.05 to all DD/MDD for all patients. Applying this technique, we performed two models per sedative. First we determined whether tracheotomy had an additive effect on the course of sedation. In the second model we added admission type and timing of tracheotomy. We determined whether these additional factors had an effect on the amount of sedation and on the course of sedation before and after tracheotomy. Differences with *p *< 0.05 were considered significant.

## Results

Of 1,788 patients admitted during the study period, 129 (7%) received a tracheotomy. Patients who received a tracheotomy on the day of admittance and patients who received a tracheotomy in another hospital before they were transmitted to our ICU were excluded from analysis, as were those who were not intubated before they received tracheotomy. This left us with 117 patients for the present analysis. Patients received a tracheotomy after a median time of nine (interquartile range 5 to 14) days from the start of MV. Patient characteristics are shown in Table 1. Basic information about the amount of sedatives used in our population is shown in Table 2.

### Summed sedative requirements

Summed sedative requirements (DD/MDD), corrected for the number of days measured, were 1.07 ± 0.93 before tracheotomy versus 0.30 ± 0.65 (*p *< 0.01) after tracheotomy for morphine, 0.84 ± 1.03 versus 0.11 ± 0.46 (*p *< 0.01) for midazolam, and 0.62 ± 1.05 versus 0.15 ± 0.45 (*p *< 0.01) for propofol. Calculated over two days before and after tracheotomy, mean summed sedative requirements were 0.43 ± 0.72 versus 0.25 ± 0.55 for morphine (*p *= 0.06), 0.20 ± 0.59 versus 0.08 ± 0.36 for midazolam (*p *= 0.06) and 0.27 ± 0.65 versus 0.11 ± 0.39 for propofol (*p *= 0.02).

### Number of patients receiving sedatives

Of all patients, 62.4% needed morphine in the week before tracheotomy, compared with 32.5% in the week after tracheotomy (*p *< 0.01). Midazolam was given to 44.4% of all patients before tracheotomy, and only 9.4% received this sedative after tracheotomy (*p *< 0.01). Propofol was used in 34.2% of the patients before tracheotomy, compared with 15.4% after tracheotomy (*p *< 0.01). The number of patients receiving all three sedatives was 17 (14.5%) before tracheotomy and 6 (5.1%) after tracheotomy (*p *< 0.01). Only 24.8% of the patients did not receive any sedatives before tracheotomy, compared with 63.2% after tracheotomy (*p *< 0.01). With regard to sedation needs in a shorter interval (two days before and two days after tracheotomy), 28.2% of the patients received morphine before tracheotomy, compared with 23.9% after tracheotomy (*p *= 0.33); 12.0% received midazolam before tracheotomy, compared with 6.0% after tracheotomy (*p *= 0.09); and 17.1% received propofol before tracheotomy, compared with 12.0% after tracheotomy (*p *= 0.11).

### Daily sedative requirements

**Figure 2:**
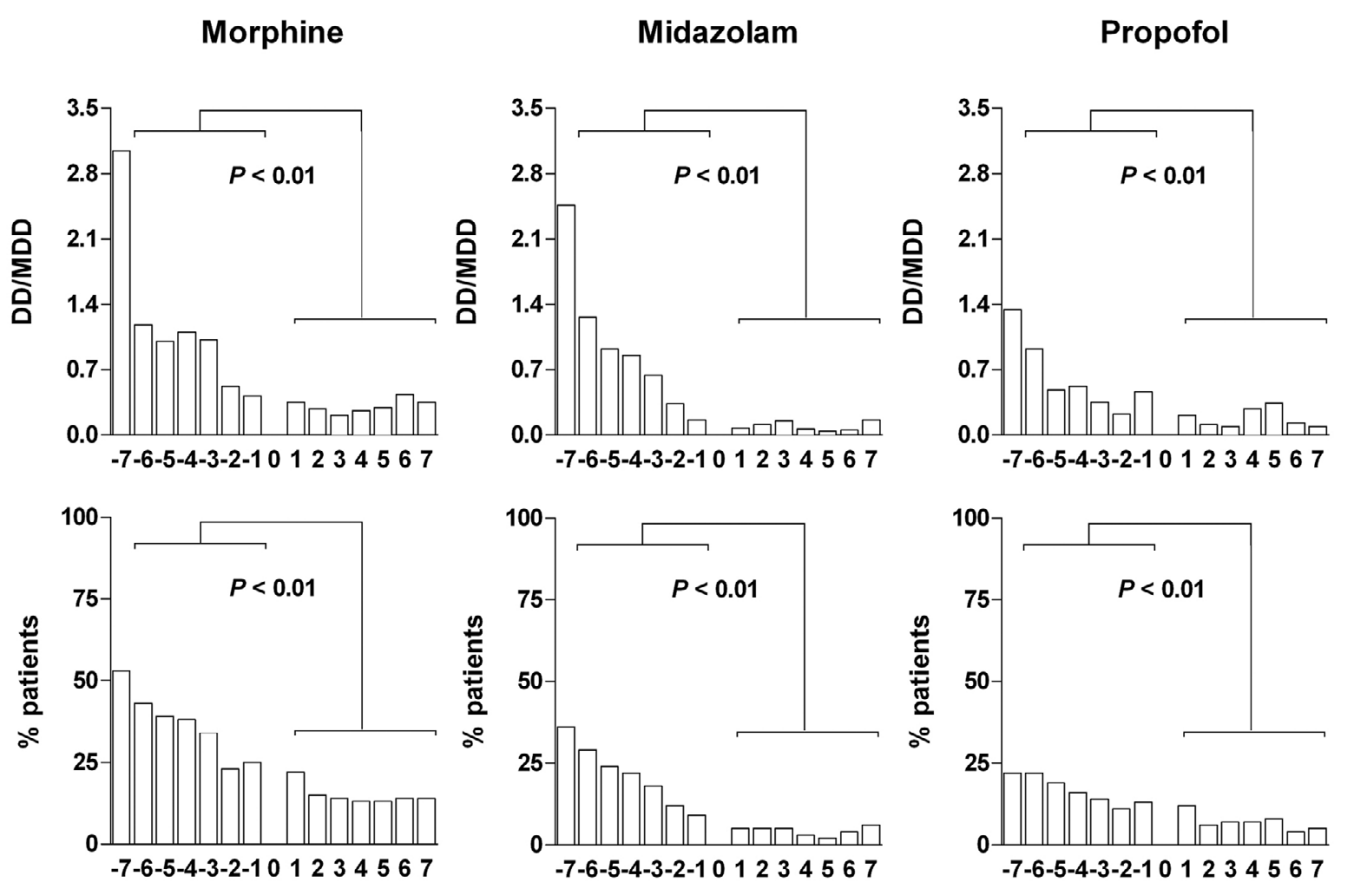
Daily administration of morphine, midazolam and propofol and percentages of patients needing these sedatives. Data are expressed as mean DD/MDD. When comparing the summed data of seven days before and after tracheotomy there was a significant difference in dosage and percentage of patients using these sedatives before and after tracheotomy (*P *< 0.01 with the Wilcoxon signed-rank test and the McNemar test). However, a repeated-measurements analysis of variance showed that, from day -7 to day -1, morphine dosage declined by 3.34 (95% confidence interval -1.61 to -6.24), midazolam dosage by 2.95 (-1.49 to -5.29) and propofol dosage by 1.05 (-0.41 to -2.01) DD/MDD (*P *< 0.01). The percentage of patients using sedatives also decreased before tracheotomy. After tracheotomy there was no further increase in decline rate, and the dosage remained stable.

Studying the course in DD/MDD from seven days before placement of tracheotomy, we found a significant decline in dosage (Figure 2). From day -7 to day -1, morphine dosage (DD/MDD) declined by 3.34 (95% confidence interval -1.61 to -6.24), midazolam dosage by 2.95 (-1.49 to -5.29) and propofol dosage by 1.05 (-0.41 to -2.01). After tracheotomy no further decrease in DD/MDD was observed; the dosage remained stable for all sedatives. The mean change in DD/MDD for morphine from day one to day seven after tracheotomy was -0.12 (-0.58 to 1.84), for midazolam it was 0.05 (-0.49 to 1.28) and for propofol it was 0.06 (-0.47 to 1.03).

The number of sedated patients over time decreased significantly toward tracheotomy, with mean reductions of 5.0%, 4.5% and 1.5% per day for morphine, midazolam and propofol, respectively. After tracheotomy there was no further change in the number of patients using sedatives (Figure 2).

### Time until tracheotomy and patient category

For all sedatives, the average DD/MDD was not significantly different between patients with early or late placement of tracheotomy. There was no difference in decline of sedation over time between early and late tracheotomy groups. In both groups the dosage remained stable after tracheotomy. Patients in the non-surgical group (*p *= 0.04) and in the acute surgery group (*p *= 0.01) had a higher average DD/MDD than patients in the elective surgery group (0.74 (0.55 to 0.98) and 0.68 (0.50 to 0.92) higher, respectively). There was no difference in the decline of midazolam over time. There was no significant difference in morphine and propofol between the different admittance type groups (*p *= 0.31 and 0.85, respectively).

## Discussion

It has been suggested that tracheotomy reduces the sedation requirements of ICU patients. This retrospective observational study shows, however, that sedation requirements were already sharply declining before tracheotomy, and that tracheotomy did not further reduce sedation needs. Similarly, the number of patients requiring sedation also decreased gradually before tracheotomy, and there was no significant difference in the number of patients receiving morphine, midazolam or propofol during the two days before tracheotomy compared with the two days after tracheotomy.

There are several limitations to our study. First, it was a retrospective analysis. Randomized trials are needed, to compare groups of patients. It is, however, very difficult to match a control group that is exactly the same as the patient group except for not receiving tracheotomy. Second, we did not collect data on sedation scores of each patient. Third, as in the study of Nieszkowska and colleagues [19], we did not implement in our sedation protocol a systematic withdrawal of sedation. This could have influenced the need for sedation before and after tracheotomy [7]. Finally, as mentioned above, this study was performed in a single-center ICU, which makes it difficult to generalize data to other ICUs.

Recent studies have shown that the use of excessive sedation prolongs the duration of MV and the length of stay in ICU, and negatively affects patient outcome [7-11,22]. It is therefore important to use strict guidelines that aim at the reduction of sedation whenever possible. Compliance with protocols in our unit is high because there is good communication between doctors and nurses. Every day the policy for each patient is discussed and nurses therefore do not have much scope for deviation from the protocols. Non-compliance with sedation protocols usually leads to oversedation [7]. This might influence the effect of tracheotomy on sedation use. A recent meta-analysis suggests that early tracheotomy shortens the duration of artificial ventilation and length of stay in the ICU [16]. In addition, Rumbak and colleagues [23] showed that early tracheotomy, as compared with prolonged endotracheal intubation, shortens the duration of MV in ICU patients and decreases the number of days spent sedated.

Nieszkowska and colleagues [19] recently described in more detail the influence of tracheotomy on the amount of sedation needed in critically ill patients. In contrast to our findings, they showed a significant decrease in sedation requirements of fentanyl and midazolam after tracheotomy. There are several possible explanations for the discrepancy between our study and that of Nieszkowska and colleagues. The first might be differences in the applied sedation protocols. In our institution the adequacy of sedation is determined by the SEDIC score. This scale was developed in 2003 by our department by combining elements of existing sedation scales, including the Riker sedation agitation scale (SAS) used in the study of Nieszkowka and colleagues. At present, no studies have been published that compare these two sedation protocols directly. The reliability of the SEDIC scale is supported by the inter-observer agreement of simultaneous but independently assessed SEDIC scores between the attending nurse and a research nurse (intra-class correlation coefficient 0.88; 95% confidence interval 0.81 to 0.92). The validity was assessed first by the observed agreement between score categories (level of stimulus and response) with a weighted kappa of 0.82, second by the ability of the SEDIC score to predict the time needed to wake up after terminating the sedative (*r*^2 ^42%), and third by the association between the Ramsay scale and the SEDIC scale, Spearman rank-order correlation coefficient of 0.74 (*p *= 0.1) [20]. In the study of Nieszkowska and colleagues, adequacy of sedation is determined every three to four hours, in contrast with every two hours in our study. It is therefore possible that with the SEDIC score deep sedation levels are noticed and corrected for at an earlier stage. In addition, the SEDIC score might be more sensitive to the detection of inappropriately high sedation in ICU patients [20].

Importantly, Nieszkowska and colleagues did not correct for individual differences in need for sedation between patients. We explicitly divided the daily dose by the mean daily dose of all days measured for each patient (DD/MDD). In this way we could overcome the possibility that some patients carried relatively more weight in the study and hence overestimated the need for sedation, especially before tracheotomy. Our timing of tracheotomy was much earlier (median nine days) than that in the study of Nieszkowska and colleagues (median 14 days). Accordingly, the timing of tracheotomy in our study cannot explain the discrepancy between the two studies. Finally, there might be an important difference in case-mix between the two studies, causing a difference in the amount of sedation needed and thereby a difference in the impact of tracheotomy on sedation. In the former study, 40% of the patients were admitted for shock and the number of cardiac patients was much higher in their study (52.7%) than in ours (13.7%). In addition, there was an important difference in ICU mortality: 30.5% versus 12.8% in our study. Accordingly, it is very difficult to generalize the results of these two single-center ICU studies because of the possible differences in sedation practice and in MV and weaning protocols between the different ICU settings.

We found that patients in the non-surgical and acute surgical groups had higher dosages of midazolam than patients in the elective surgical group, but there was no difference in the rate of decline before or after tracheotomy between patient groups. Importantly, we found no influence of timing of tracheotomy on sedation requirements. Consequently, the high decline in sedation before tracheotomy cannot be explained by the timing of tracheotomy. In both early and late tracheotomy patients the dosage remained stable after tracheotomy.

## Conclusion

We found no convincing support for the hypothesis that tracheotomy reduced the sedation needs of critically ill patients in our ICU. In our center, using a strict sedation protocol, sedation requirements were already sharply declining before tracheotomy was performed.

## Key messages

• 	Tracheotomy did not reduce the sedation needs of critically ill patients in our ICU.

• 	Additional studies, specifically in centers with different case-mixes and different sedation protocols, will be necessary before these results can be generalized.

## Abbreviations

DD = daily dose; FiO_2 _= fraction of inspired oxygen; ICU = intensive care unit; MDD = mean daily dose; MV = mechanical ventilation; PEEP = positive end-expiratory pressure; SEDIC = Sedation Intensive Care.

## Competing interests

The authors declare that they have no competing interests.

## Authors' contributions

DPV participated in the analysis and interpretation of the data and in drafting the manuscript. DAD contributed to the conception and design of the study and revision of the manuscript. JCK was involved in the design and statistical analysis of the study. JMB and MBV were involved in drafting and revising the manuscript critically for important intellectual content. MJS conceived and coordinated the study and was involved in the interpretation of the data and in revision of the manuscript. All authors read and approved the final manuscript.

## Figures and Tables

**Table 1:** Demographic data

Parameter	Patients with tracheostomy included in study
Number of patients	117
Age (years)	57.6 (46.3–71.8)
Gender, male	78 (66.7)
APACHE II score	21.0 (16.0–26.0)
SAPS II score	44.0 (35.0–57.0)
ICU mortality	15 (12.8)
Hospital mortality	39 (33.3)
LOS in ICU (days)	17.8 (11.9–30.1)
Time until tracheotomy (days)	9.0 (5.0–14.0)
Admission diagnosis	
Medical	20 (17.1)
Surgical	36 (30.8)
Neurology/neurosurgery	29 (24.8)
Cardiopulmonary surgery	12 (10.3)
Cardiology	16 (13.7)
Admission type	
Non-surgical	58 (49.6)
Acute surgical	32 (27.4)
Elective surgical	27 (23.0)

Where ranges are shown, results are medians (interquartile range); results followed by single numbers in parentheses are *n *(%). APACHE, Acute Physiology and Chronic Health Evaluation; SAPS, Simplified Acute Physiology Score; ICU, intensive care unit; LOS, length of stay.

**Table 2:** Basic data on sedation dose

Parameter	7 days before tracheotomy	7 days after tracheotomy	2 days before tracheotomy	2 days after tracheotomy
Morphine				
Patients on morphine (%)	62.4	32.5	28.2	23.9
Mean dose (mg/day)	21.5 ± 30.2	5.7 ± 16.6	5.6 ± 13.8	2.7 ± 8.3
Maximum dose (mg/day)	132.5	120.0	85.0	67.7
Midazolam				
Patients on midazolam (%)	44.4	9.4	12.0	6.0
Mean dose (mg/day)	28.6 ± 66.3	6.5 ± 45.7	6.5 ± 23.7	2.6 ± 15.0
Maximum dose (mg/day)	415.9	478.6	160.3	137.1
Propofol				
Patients on propofol (%)	34.2	15.4	17.1	12.0
Mean dose (mg/day)	448.25 ± 957.25	171.54 ± 667.92	283.79 ± 823.69	175.67 ± 687.02
Maximum dose (mg/day)	5,000	5,455.14	5,000	4,900

The table presents data that have not been adapted for analysis. Where errors are shown, results are means ± SD.
